# Nail Mycosis Fungoides: A New Case

**DOI:** 10.7759/cureus.19913

**Published:** 2021-11-26

**Authors:** Fouzia Hali, Ntihebuwayo Jean Berchmans, Farida Marnissi, El Kebir Asma, Soumiya Chiheb

**Affiliations:** 1 Department of Dermatology, University Hospital Center Ibn Rochd, University Hassan II, Casablanca, MAR; 2 Central Pathological Anatomy Laboratory, University Hospital Center Ibn Rochd, University Hassan II, Casablanca, MAR

**Keywords:** biopsy of the nail matrix and nail, pilotropic, nail lymphoid infiltration, nail changes, mycosis fungoides

## Abstract

Ungueotropic mycosis is a rare form of mycosis fungoides. We present the case of a 32-year-old female patient with advanced tumor stage mycosis fungoides, presenting a phanerial involvement with lymphoid infiltration of the nails and scalp confirmed by histology and immunohistochemistry.

## Introduction

Ungueotropic mycosis is defined as a rare variant of mycosis fungoides (MF) with predominantly nail involvement. Nail changes in primary cutaneous T-cell lymphoma have been rarely described. Nail involvement is seen in mycosis fungoides (MF), usually at an advanced stage, as well as in true Sezary syndrome. Only eight cases of mycosis fungoides with histologically and immunohistochemically confirmed nail involvement have been reported in the literature [1-4].

We report a case of advanced mycosis fungoides with nail involvement and palmar keratosis hands as a starting point.

## Case presentation

**Figure 1:**
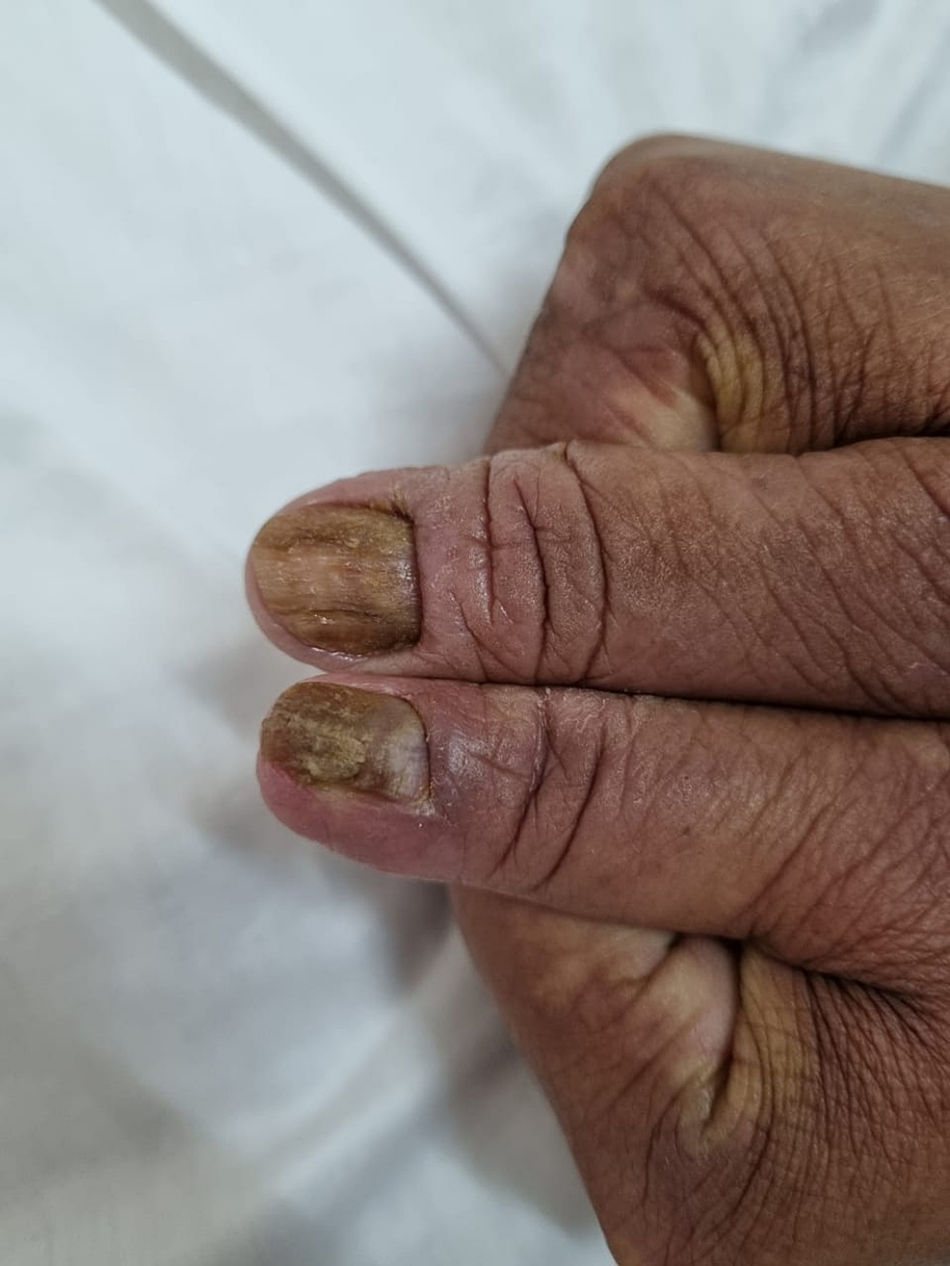
Xanthopachyonychia, trachyonychia, and perionyxis of both thumbs.

A 32-year-old female patient presented with pruritic palmar keratosis of the hands for two years, followed one year later by the appearance of infiltrated and pruritic patches of skin and onychodystrophy of the fingernails concomitant with diffuse alopecia of the scalp. Examination of the nails showed xanthopachyonychia, trachyonychia, and perionyxis of both thumbs (Figure 1).

**Figure 2:**
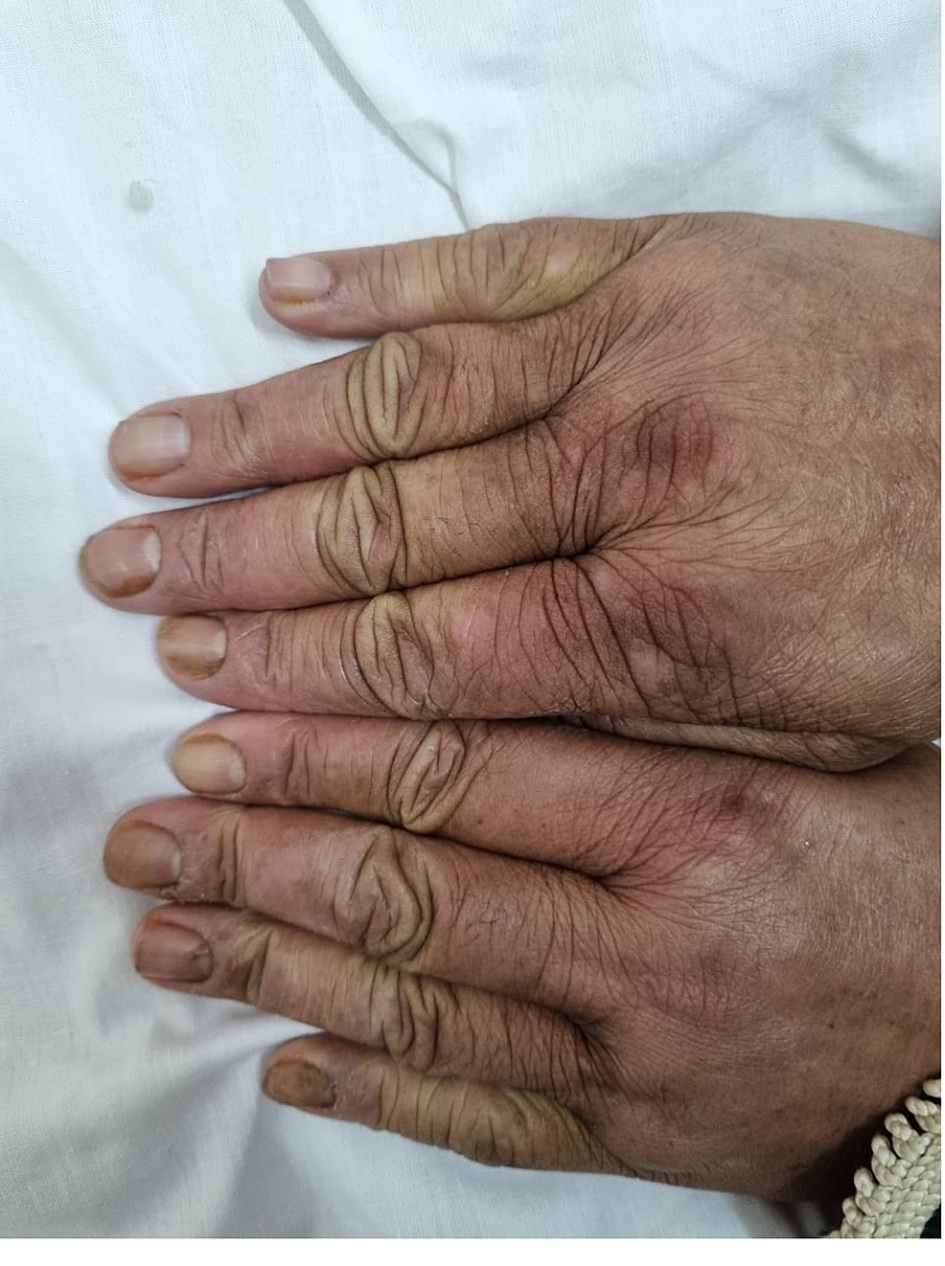
Trachyonychia and lateroproximal xanthonychia of the right index finger and the left ulnar.

Trachyonychia and lateroproximal xanthonychia of the right index finger and the left ulnar (Figure 2).

**Figure 3:**
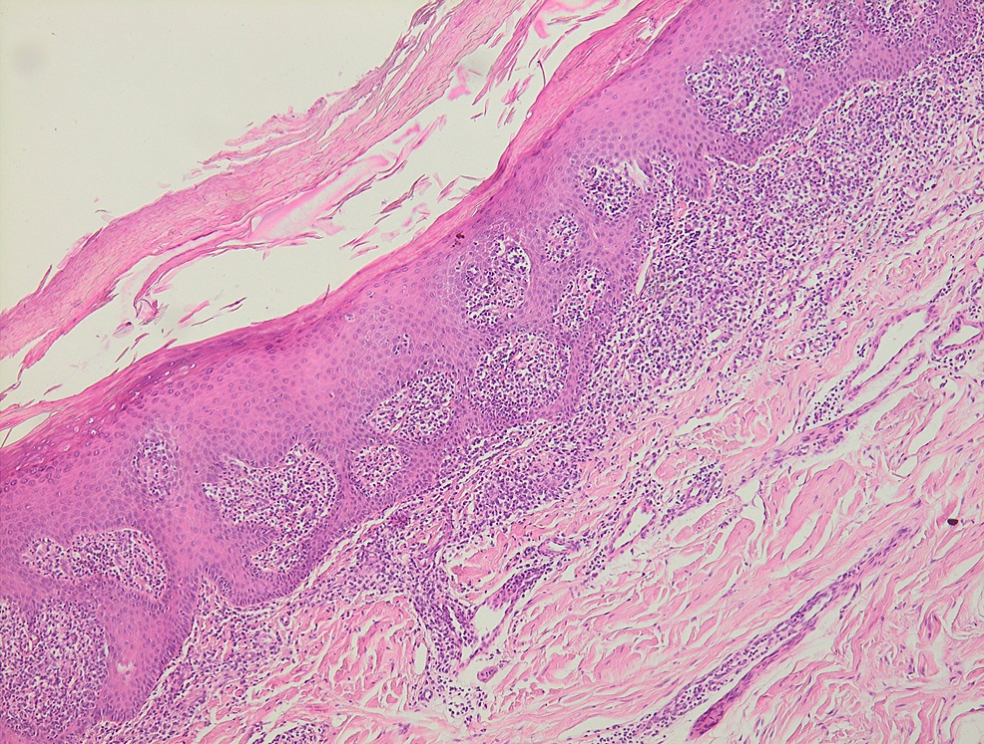
Histological biopsy of the nail matrix and nail bed showed a banded lymphocytic proliferation with focal epidermotropism.

Histological biopsy of the nail matrix and nail bed showed a banded lymphocytic proliferation with focal epidermotropism. Cell size was small to medium with angular or rounded nuclei, associated with histiocytes (Figure 3).

**Figure 4:**
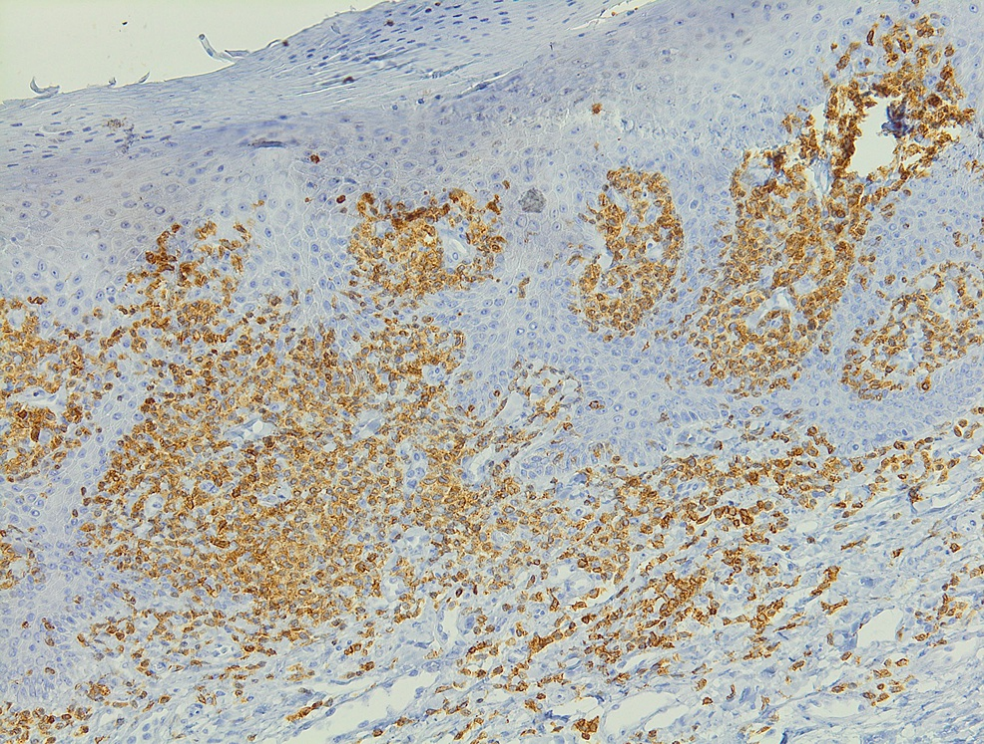
Immunohistochemistry showed an atypical lymphocytic infiltrate strongly expressing CD3.

**Figure 5:**
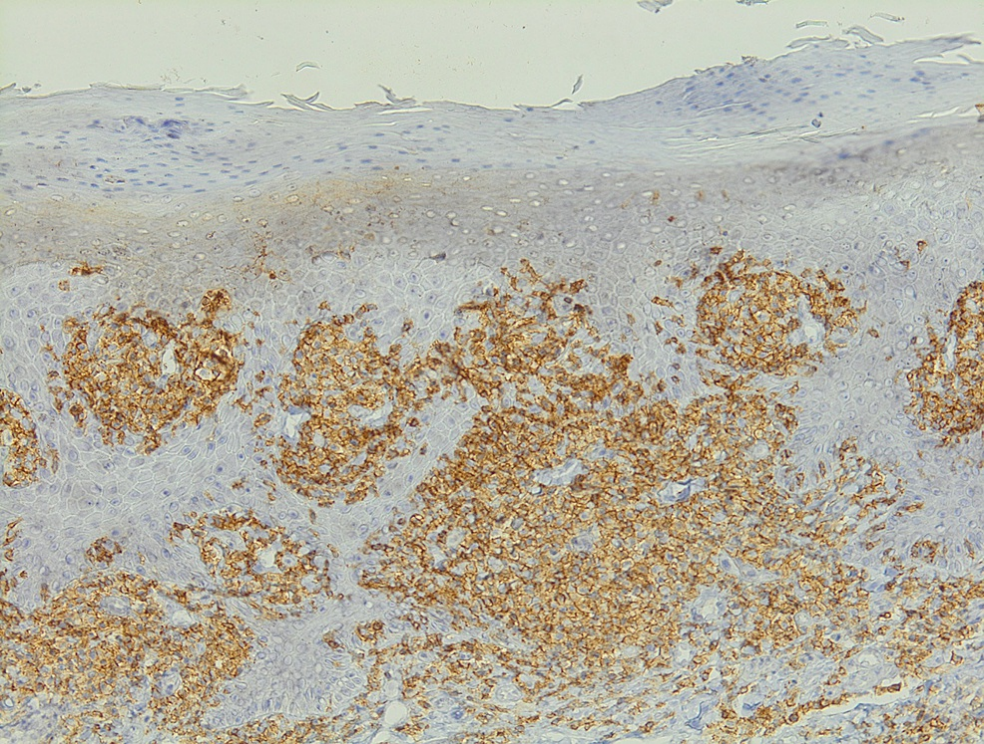
Immunohistochemistry showed an atypical lymphocytic infiltrate strongly expressing CD4.

Immunohistochemistry showed an atypical lymphocytic infiltrate strongly expressing CD3 and CD4 in a superimposable manner (Figures 4, 5).

CD8 was negative. The histological and immunohistochemical conclusion was in favor of a nail mycosis fungoides without signs of transformation. Biopsy of an infiltrated patch of skin, alopecia, and forehead nodule concluded pilotropic mycosis fungoides. Bone marrow biopsy for bone marrow invasion, cervical adenopathy for lymph node involvement, blood smear for sezary cells in the blood were all normal. The biological workup showed eosinophilic leukocytosis up to 20,300 cells/m^3^, IgE 307.09 IU/mL, lactate dehydrogenase 467 IU/L, C-reactive protein 22 mg/L, liver and kidney function within normal limits. CT scans of the lungs, abdomen, and pelvis were normal. A diagnosis of pilotropic mycosis fungoides tumor stage IIB: T3N1M0B0 with nail involvement was made. Pyrimidine analogue and platinum-based antineoplastic chemotherapy were initiated. The evolution was marked by a slight disinfiltration of the plaques and nodules and a slight decrease of the perinonxys with slight improvement of the trachyonychia and xanthonychia of the right index finger and the left ulnar after three courses of chemotherapy.

## Discussion

Mycosis fungoides with histologically and immunohistochemically confirmed nail involvement has been rarely described in the literature [4,5]. Its incidence is poorly known, and only eight cases confirmed by nail biopsy have been reported [1,4,6,7]. In all published reports, the types and stages of mycosis fungoides varied from case to case. Nail changes ranged from simple yellowish discoloration to slowed growth, nail thickening and increased curvature, and onychomadesis. Previously reported types of mycosis fungoides with nail involvement confirmed by histology and immunohistochemistry included folliculotropic, pilotropic, and palmoplantar mycosis fungoides [1,8,9]. Nail involvement has also been observed in advanced-stage mycosis fungoides as well as in true Sézary syndromes [4,6,7,10,11]. Paronychia, trachyonychia, onycholysis, and subungual hyperkeratosis have been described as a direct consequence of folliculotropic mycosis infiltration affecting the nails [7]. Some nail manifestations were considered specific to mycosis fungoides. These included yellowish discoloration of the nails, slow growth, thickening, onycholysis, onychomadesis, subungual hyperkeratosis, and pterygium formation [4,5]. The nail changes in mycosis fungoides are thought to be related to the abnormal production of T cells in the skin, which are responsible for the prolonged growth arrest and general nail impairment, apart from any other factors and diseases that may affect nail growth, morphology, and appearance. Another thesis suggested that the clinical changes of the nails were related either to a non-specific effect of chronic erythroderma as in the case of Sézary syndrome or to an infiltration of T lymphocytes in the nail apparatus [7]. Lichen planus is thought to be one of the precursor dermatoses of mycosis fungoides [7]. To confirm nail involvement in mycosis fungoides, biopsies should include the nail bed and matrix [7-9]. The frequency of nail involvement may be underestimated because skin biopsies showing erythema and scaling around the nail plate with clinically significant nail changes have not revealed any evidence of mycosis fungoides [5]. To our knowledge, we report the first case of mycosis fungoides with nail involvement that presented with concomitant onychodystrophy and diffuse alopecia of the scalp. Treatment of nail involvement in mycosis fungoides is not well codified because of the limited number of cases reported. Treatments have included chlorambucil, gemtabicin, puvatherapy, and electron beam radiation therapy with partial responses or complete remissions [5,7].

## Conclusions

Our observation illustrates a rare presentation of histologically and immunohistochemically confirmed nail mycosis fungoides. The coexistence of onychodystrophy and alopecia should be recognized and considered in all patients with suspected mycosis fungoides. Matrix and nail bed biopsy should be routinely offered to confirm nail involvement in mycosis fungoides. Further studies are needed to determine whether the involvement of the dander during cutaneous T-cell lymphoma can provide useful information about disease progression before or during treatment.
